# Rotenone-Induced 4-HNE Aggresome Formation and Degradation in HL-1 Cardiomyocytes: Role of Autophagy Flux

**DOI:** 10.3390/ijms23094675

**Published:** 2022-04-23

**Authors:** Sudha Sharma, Foram Patel, Hosne Ara, Ezra Bess, Alika Shum, Susmita Bhattarai, Utsab Subedi, Daquonte Sanard Bell, Md. Shenuarin Bhuiyan, Hong Sun, Ines Batinic-Haberle, Manikandan Panchatcharam, Sumitra Miriyala

**Affiliations:** 1 Department of Cellular Biology and Anatomy, Louisiana State University Health Sciences Center, Shreveport, LA 71103, USA; sudha.sharma@lsuhs.edu (S.S.); forampatel@live.com (F.P.); hosne.ara@lsuhs.edu (H.A.); eub001@lsuhs.edu (E.B.); alika.shum@lsuhs.edu (A.S.); susmita.bhattarai@lsuhs.edu (S.B.); utsab.subedi@lsuhs.edu (U.S.); sanardbell@yahoo.com (D.S.B.); hong.sun@lsuhs.edu (H.S.); 2 Department of Pathology and Translational Pathobiology, Louisiana State University Health Sciences Center, Shreveport, LA 71103, USA; shenu.bhuiyan@lsuhs.edu; 3 Department of Radiation Oncology, Duke University School of Medicine, Durham, NC 27710, USA; ibatinic@duke.edu

**Keywords:** oxidative stress, rotenone, autophagy, autophagosome, Mn porphyrin

## Abstract

Reactive oxygen species (ROS) cause oxidative stress by generating reactive aldehydes known as 4-hydroxynonenal (4-HNE). 4-HNE modifies protein via covalent adduction; however, little is known about the degradation mechanism of 4-HNE-adducted proteins. Autophagy is a dynamic process that maintains cellular homeostasis by removing damaged organelles and proteins. In this study, we determined the role of a superoxide dismutase (SOD) mimetic MnTnBuOE-2-PyP^5+^ (MnP, BMX-001) on rotenone-induced 4-HNE aggresome degradation in HL-1 cardiomyocytes. A rotenone treatment (500 nM) given for 24 h demonstrated both increased ROS and 4-HNE aggresome accumulation in HL-1 cardiomyocytes. In addition, cardiomyocytes treated with rotenone displayed an increase in the autophagy marker LC3-II, as shown by immunoblotting and immunofluorescence. A pre-treatment with MnP (20 µM) for 24 h attenuated rotenone-induced ROS formation. An MnP pre-treatment showed decreased 4-HNE aggresomes and LC3-II formation. A rotenone-induced increase in autophagosomes was attenuated by a pre-treatment with MnP, as shown by fluorescent-tagged LC3 (tfLC3). Rotenone increased tubulin hyperacetylation through the ROS-mediated pathway, which was attenuated by MnP. The disruption of autophagy caused HL-1 cell death because a 3-methyladenine inhibitor of autophagosomes caused reduced cell death. Yet, rapamycin, an inducer of autophagy, increased cell death. These results indicated that a pre-treatment with MnP decreased rotenone-induced 4-HNE aggresomes by enhancing the degradation process.

## 1. Introduction

Cardiovascular disease is the leading cause of death worldwide. One of the major factors in the pathogenesis of cardiovascular disease is oxidative stress; thus, the mechanism of this stress requires a better understanding [1]. A major site for oxidative stress is the mitochondria due to the production of reactive oxygen species (ROS) from complexes I and III of the electron transport chain [2]. ROS then react with lipids, causing lipid peroxidation. A stable, yet deleterious, aldehyde produced by lipid peroxidation is 4-hydroxynonenal (4-HNE). It has been well-established that 4-HNE reacts with protein and causes its modification [3,4]. However, the modification of proteins by 4-HNE and the sequential degradation mechanism have been not been well-studied. In light of the strong connection between mitochondrial ROS and cardiac disease, scientists are interested in discovering a way to detoxify mitochondrial ROS to ameliorate the disease outcomes. One method of interest among the scientific community is to use a mitochondrially-based manganese superoxide dismutase mimetic [5].

Autophagy is a basic cellular process that plays a vital role in maintaining cellular homeostasis. This is achieved by removing misfolded or aggregated proteins, damaged cellular organelles, and intracellular pathogens [6]. Decreases in the autophagy process have been linked to several pathologies, including neurodegenerative, liver, and cardiac diseases as well as cancer [7]. There are three main types of autophagy: macroautophagy, microautophagy, and chaperone-mediated autophagy. Macroautophagy differs from the other two autophagy processes with its unique formation of an autophagosome. The autophagosome acts as a sequestering vesicle for cellular waste, which ultimately fuses with a lysosome in order to induce waste degradation within the vesicle. This process requires the involvement of several proteins, including ATG-12, ATG-5, Beclin-1, and microtubule-associated protein (MAP) light chain 3 (LC3) [6]. Microtubules play a vital role in the transportation of cargo within the cell. The acetylation of microtubules enables an autophagosome fusion with the lysosome, making it an essential part of the autophagy process [8]. Rotenone, a mitochondrial respiratory complex I inhibitor, has been used in previous studies to better understand the role of autophagy in the disease processes [9,10]. Rotenone-mediated oxidative stress leads to 4-HNE adduction with several proteins to form aggresomes. It has been shown that the degradation of aggresomes specifically occurs through the autophagy process, which involves autophagosome and autolysosome formation [11]. The disruption of autophagy leads to theaccumulation of these aggresomes, leading to the cytotoxicity and cell death [11].

This study explores the role of the SOD mimetic, MnTnBuOE-2-PyP^5+^ (MnP) on rotenone-induced 4-HNE aggresome formation and the aggresome degradation process via autophagy in cardiomyocytes. Rotenone was used to induce ROS formation, leading to an excess production of 4-HNE. Protein aggregation was achieved following the modification of proteins by 4-HNE. It is known that a reduction in the autophagy degradation process causes an overwhelming amount of protein aggregates to remain inside the cells. Therefore, we used an MnP-based SOD mimetic, which preferentially accumulates in the mitochondria to ameliorate the aggregation.

## 2. Results

### 2.1. Rotenone Induces the Production of ROS in a Dose-Dependent Manner

**Figure 1:**
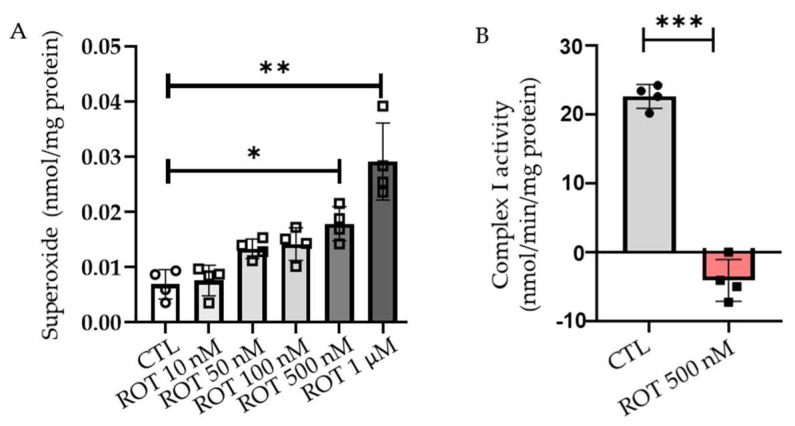
Intracellular superoxide production in HL-1 cells after treatments with various concentrations of rotenone. (**A**) The level of intracellular superoxide in HL-1 cells was measured by HPLC analysis of DHE oxidation product after treatment with 10 nM-1 µM rotenone (ROT) for 24 h compared with the control (CTL) (*n* = 4). (**B**) Mitochondrial complex I activity was measured upon rotenone 500 nM (ROT) treatment for 24 h. Data are plotted as mean ± SD. * *p* < 0.05; ** *p* < 0.01; *** p < 0.001.

Rotenone, an inhibitor of complex I of the electron transport chain, has been shown to increase ROS production in ARPE-19 [11], HL-60 [12], and HT22 cells [13]. We tested the ability of rotenone to produce ROS at both intracellular and mitochondrial levels in HL-1 cardiomyocytes. To define the dosage required for rotenone to produce ROS, we incubated cells with different concentrations (10 nM, 50 nM, 100 nM, 500 nM, and 1 µM) of rotenone for 24 h. The increase in rotenone concentration from 10 nM to 1 µM over 24 h showed a significant rise in intracellular ROS production, starting at a 500 nM concentration. At 500 nM of rotenone, there was an almost two-fold increase in ROS production; 1 µM of rotenone produced more than a three-fold increase in intracellular ROS production compared with the control (Figure 1A). Rotenone inhibits the electron transfer from the iron-sulfur centers in complex I to ubiquinone. This, in turn, causes the incomplete transfer of an electron to oxygen, leading to the production of ROS [14]. Therefore, we measured the ability of rotenone to cause the inhibition of complex I activity in HL-1 cardiomyocytes. We observed a significant reduction in complex I activity following a treatment of 500 nM rotenone for 24 h (Figure 1B). Taken together, the results indicated that rotenone produced ROS by the inhibition of complex I activity in HL-1 cardiomyocytes.

### 2.2. Rotenone-Induced ROS Lead to 4-HNE Inclusions Attenuated by MnTnBuOE-2-PyP^5^^+^ (MnP)

**Figure 2:**
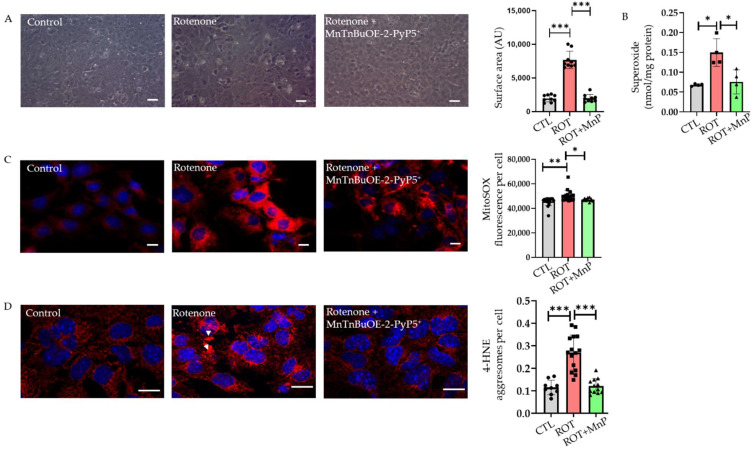
Alternation of cell morphology, superoxide production, and 4-HNE aggresome formation by rotenone treatment attenuated with MnTnBuOE-2-PyP^5+^ pre-treatment. (**A**) Cellular morphological changes were visualized using a phase-contrast microscope. An increase in cell surface area was observed in HL-1 cells treated with rotenone (500 nM) for 24 h (scale bar, 50 µm). Right panel, quantification of cell surface area. (**B**) The levels of intracellular superoxide production in control (CTL), rotenone (ROT), and rotenone+MnTnBuOE-2-PyP^5+^ (ROT + MnP)-treated HL-1 cells was evaluated by HPLC analysis of DHE oxidation product (*n* = 4). (**C**) Mitochondrial superoxide formation was measured with a MitoSOX fluorescence probe. Representative images of MitoSOX fluorescence were obtained using fluorescence microscopy from control, rotenone, and rotenone+ MnTnBuOE-2-PyP^5+^-treated HL-1 cells (scale bar, 10 µm) (*n* = 5). (**D**) Representative image of 4-HNE fluorescence in HL-1 cells challenged with rotenone (500 nM) alone or cells pre-treated with MnP (20 µM) 24 h before rotenone (500 nM) treatment (scale bar, 4 µm). Right panel, statistical analysis of 4-HNE aggresomes per cell (*n* = 5). Arrow indicates 4-HNE aggresomes. Data are plotted as mean ± SD. * *p* < 0.05; ** *p* < 0.01; *** *p* < 0.001.

MnTnBuOE-2-PyP^5+^ (MnP) is a SOD mimetic, which preferentially accumulates in the mitochondria where it mimics MnSOD. It has been reported that MnP reduces ROS-based cellular toxicity. This SOD mimetic is currently undergoing early clinical trials to reduce radiation- and chemotherapy-induced oxidative stress [15]. The use of MnP has been shown to be effective in other cell lines. The morphology of HL-1 cells under the control settings, or treated with rotenone, or rotenone + MnP was observed under phase-contrast microscopy. The cells treated with 500 nM rotenone for 24 h increased in surface area. However, this undesired effect was rescued in cells pre-treated with MnP (Figure 2A).

The treatment with 20 µM MnP for 24 h significantly reduced the intracellular superoxide produced by the rotenone treatment (Figure 2B). A MitoSOX probe was used to measure ROS production in the mitochondria of HL-1 cardiomyocytes. The results showed that at a concentration of 20 µM, an MnP pre-treatment for 24 h attenuated the rise in mitochondrial ROS after the rotenone treatment (Figure 2C). 4-hydroxynonenal (4-HNE) is a byproduct of lipid peroxidation and is considered to be a marker of oxidative stress [3]. The immunofluorescence labeling of the HL-1 cells showed diffused 4-HNE staining at a basal level compared with the rotenone-treated cells where 4-HNE was stained within a dense structure of aggresomes. A quantitative analysis of the aggresomes showed a more than two-fold rise in the cells treated with rotenone compared to the basal level. Furthermore, MnP attenuated the formation of 4-HNE aggresomes (Figure 2D). Rotenone-mediated ROS generation overwhelmed the cellular antioxidant capacity and led to the accumulation of 4-HNE aggresomes.

### 2.3. Characterization of Rotenone-Induced 4-HNE Aggresomes

**Figure 3:**
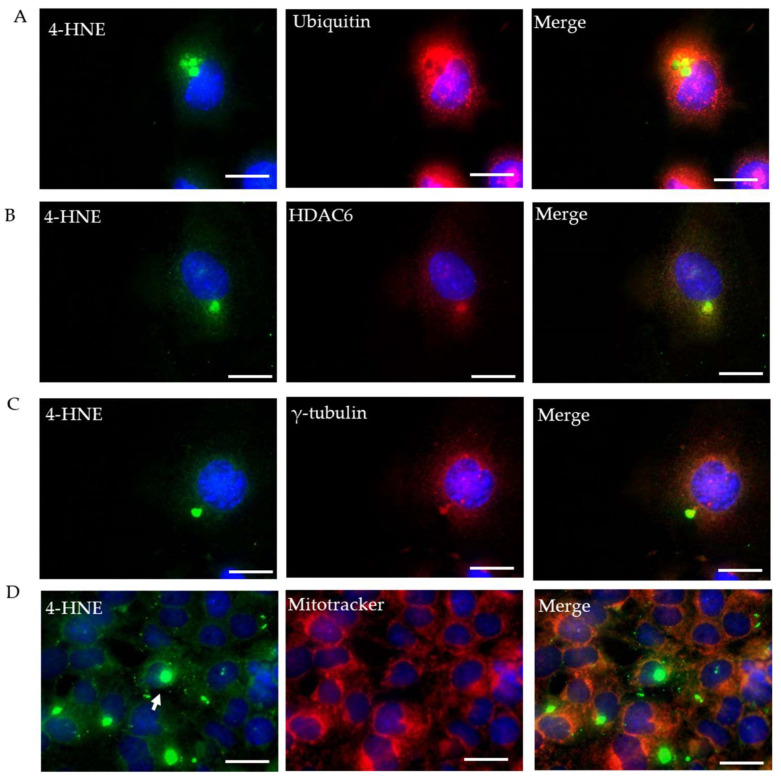
Characterization of rotenone-induced 4-HNE aggresomes. HL-1 cells were treated with rotenone (500 nM) for 24 h then fixed and processed for double immunostaining. (**A**–**C**) Fluorescent images of 4-HNE aggresome (green) co-localization with autophagy markers ubiquitin, HDAC6, and γ-tubulin (red), respectively. (**D**) Co-immunofluorescence of 4-HNE with mitotracker (mitochondria). The nucleus is stained with DAPI (blue). Arrow indicates 4-HNE aggresomes (scale bar, 4 µm).

For the further characterization of the 4-HNE aggresomes, we co-immunostained 4-HNE with three proteins contributing to the autophagy process: ubiquitin, γ-tubulin, and HDAC6. All three stained proteins exhibited co-localization with the 4-HNE aggresomes. We also co-immunostained 4-HNE with a mitotracker to determine the location of the 4-HNE aggresomes within the cell. The mitotracker indicated that the 4-HNE aggresomes localized outside the mitochondria (Figure 3A–D). Misfolded proteins such as ubiquitin, HDAC6, and γ-tubulin were deposited within the rotenone-induced 4-HNE aggresomes, signifying a few complications in the autophagy process.

### 2.4. Rotenone Disrupts Autophagy Flux

**Figure 4:**
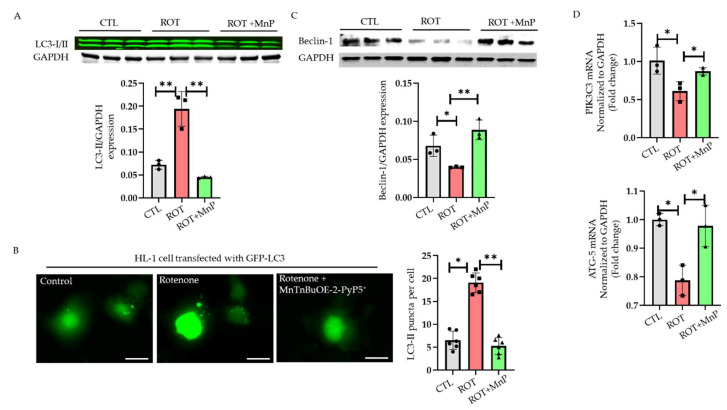
Effects of rotenone on autophagy pathway in HL-1 cells. HL-1 cells were treated with rotenone (ROT) alone or treated with rotenone following pre-treatment with MnP (20 µM) for 24 h. (**A**) Cells were processed for Western blot analysis using antibody against LC3-II /GAPDH and I (*n* = 3). (**B**) Representative fluorescence images of GFP-LC3 puncta following treatment with rotenone (ROT) or rotenone + MnP (ROT + MnP) (scale bar, 4 µm). Right panel, quantification of GFP-LC3 puncta per cell (*n* = 6). (**C**) Western blot analysis of autophagy marker Beclin-1 where GAPDH was used as a loading control (CTL) (*n* = 3). (**D**) Taqman qPCR assay used to measure mRNA levels of PIK3C3 and ATG-5. GAPDH was used as a loading control (CTL) (*n* = 3). All values are mean ± SD. * *p* < 0.05; ** *p* < 0.01.

Autophagy involves the maintenance of the proteostasis of the cell by degrading unwanted or misfolded proteins. The main characteristics of early autophagy include the lipidation of LC3-I to LC3-II and the recruitment of LC3-II to the growing autophagosome. This process also depends upon several proteins, including ATG-12, ATG-5, and Beclin-1 [16]. When the HL-1 cardiomyocytes were subjected to a rotenone treatment (500 nM), a significant increase in endogenous LC3 was observed compared with the control. However, the increase in endogenous LC3 was entirely attenuated by a pre-treatment with MnP (20 µM) for 24 h (Figure 4A). To observe LC3-II puncta in the cell, we transfected the HL-1 cardiomyocytes with adenovirus GFP-LC3 and allowed 24 h for a viral expression within the cell. The cells were challenged with 500 nM rotenone for 24 h. When the cells were observed under a fluorescent microscope, an increase in the number of LC3 puncta was seen in the rotenone-treated cells compared with the control group. Cells pre-treated with MnP showed a reduced number of puncta compared with those not pre-treated (Figure 4B). We also checked for Beclin-1 protein expression, which was reduced following the rotenone treatment (Figure 4C). This reduction, however, was attenuated in the cells pre-treated with MnP. There was a reduction in the mRNA expression of other autophagy-related proteins such as PIK3C3 and ATG-5, which was attenuated with an MnP treatment (Figure 4D).

**Figure 5:**
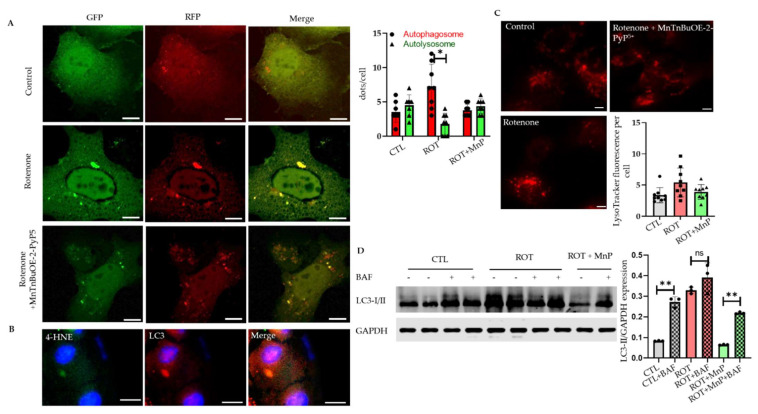
Rotenone-induced autophagy flux disruption attenuated by MnP. HL-1 cells transfected with tfLC3 treated with rotenone alone or with rotenone following pre-treatment with MnP. (**A**) Cells were fixed and images were obtained with a fluorescence microscope. The number of puncta per cell was counted using ImageJ software (*n* = 4) (scale bar, 10 µm). Right panel, quantification of dots per cell. (**B**) Representative images of double immunofluorescence staining of 4-HNE (green) and LC3 (red). (**C**) Representative image of lysotracker Deep Red staining in HL-1 cells treated with rotenone (500 nM, 24 h) or MnP (20 µM, 24 h) followed by rotenone (500 nM, 24 h). Right panel, quantification of lysotracker fluorescence intensity (*n* = 4) (scale bar, 10 µm). (**D**) Western blot image of LC3-I/II from mouse adult cardiac myocytes. Cells were incubated with bafilomycin (BAF; 50 nM) for 2 h before the endpoint. All values are mean ± SD. * *p* < 0.05; ** *p* < 0.01.

To determine whether the increase in autophagosome was due to the induction of the autophagy process or the disruption of degradation, we transfected a vector with a tandem fluorescent-tagged LC3 (tfLC3) reporter. Tandem fluorescent-tagged LC3 is effective in the simultaneous assessment of autophagosome formation and its degradation in the lysosome. Using this process, autophagosomes labeled with green and red probes yield yellow puncta whereas autolysosomes labeled with red probes yield red puncta within the cell [17]. After 24 h of incubation, the cells challenged with rotenone showed a higher number of autophagosomes than autolysosomes, indicating that the issue was a disruption of autophagy rather than induction. MnP usage showed results comparable with the control group where autolysosomes were present equal to those of autophagosomes (Figure 5A). We also determined the co-localization of 4-HNE aggresomes with LC3 using co-immunofluorescence (Figure 5B). To verify that the lysosomal function was not affected by the rotenone treatment, we incubated HL-1 cells with LysoTracker, a red fluorescent dye that indicates the lysosomal pH. No significant difference in lysotracker fluorescence was observed between the groups (Figure 5C). To further verify the effect of rotenone on the disruption of autophagy, we treated adult cardiomyocytes with bafilomycin, a lysosome inhibitor that blocks the fusion of autophagosomes with autolysosomes [18]. In the absence of rotenone, we observed a more remarkable increase in LC3-II with a bafilomycin treatment. Although we observed a significant increase in the LC3-II expression following the rotenone treatment compared with the control, the bafilomycin treatment did not produce any further increase in the LC3-II expression in the rotenone-treated cells, suggesting that rotenone disrupted autophagy flux at the terminal step. In the MnP-treated group, a similar effect was seen to that of the control cells (Figure 5D). These results indicated that rotenone-mediated mitochondrial ROS disrupted autoplagy flux.

### 2.5. Rotenone-Stimulated Tubulin Hyperacetylation Causes Autophagy Disruption

**Figure 6:**
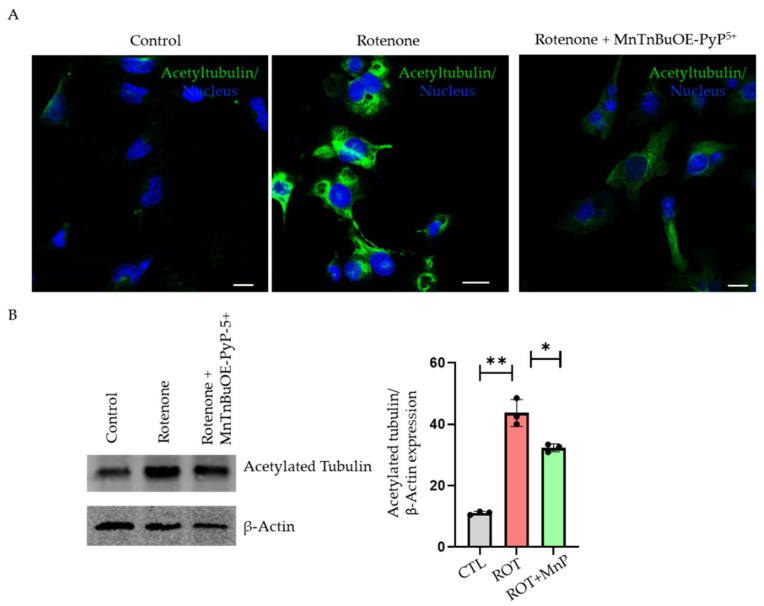
Autophagy flux disrupted by tubulin hyperacetylation. (**A**) Representative images of acetylated tubulin in HL-1 cells from control (CTL), rotenone (ROT) (500 nM, 24 h), and rotenone+MnTnBuOE-2-PyP^5+^ (ROT) groups. Immunofluorescence of acetylated tubulin increased in rotenone-treated cells and fluorescence was reduced in cells pre-treated with MnP. (**B**) Representative Western blot of HL-1 cell lysate using anti acetylated tubulin antibody. The expression of acetylated tubulin was normalized with β-Actin (*n* = 3) (scale bar, 20 µm). All values are mean ± SD. * *p* < 0.05; ** *p* < 0.01.

Tubulin acetylation is important for the stability of the microtubule. However, tubulin hyperacetylation has been shown to disrupt autophagy flux in a few conditions [19]. We used both immunofluorescence and Western blot methods to show tubulin hyperacetylation. In immunofluorescence, the rotenone-treated cells showed increased acetylation compared with the control. MnP was able to rescue the effect of the rotenone on tubulin acetylation (Figure 6A). A Western blot with acetylated tubulin showed an increased expression upon rotenone treatment, which was attenuated upon MnP pre-treatment (Figure 6B). This suggested that tubulin hyperacetylation following a rotenone treatment was mediated by ROS.

### 2.6. Role of Disrupted Autophagy Flux in Cell Viability

**Figure 7:**
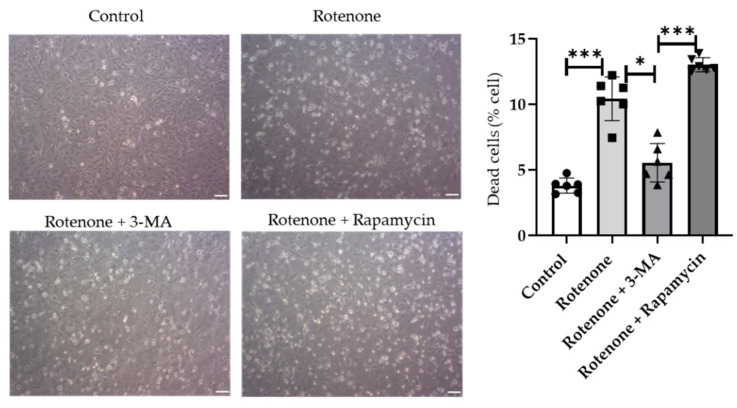
Rotenone-induced cell death is caused by disrupted autophagy flux. Cell visualized using a phase-contrast microscope (scale bar, 50 µm). The bar graph shows cell death induced by rotenone in the presence of 3-MA (autophagy inhibitor) or rapamycin (autophagy inducer) and was evaluated by the Evans blue exclusion method (*n* = 4) (scale bar, 20 µm). Data shown are the mean  ±  SD. * *p* < 0.05; *** *p* < 0.001.

To determine how the disruption of autophagy flux causes increased cell death, we treated one group of cells with a known autophagy inhibitor, 3-MA, and another group of cells with a canonical autophagy inducer, rapamycin. The cells were treated for 30 min before the rotenone treatment. The 3-MA pre-treatment showed decreased cell death whereas the rapamycin pre-treatment increased cell death (Figure 7).

## 3. Discussion

Autophagy is a dynamic process that is essential for the turnover of damaged organelles and protein degradation. Autophagy begins with the formation of a double membrane structure called an autophagosome, which ultimately fuses with a lysosome to degrade damaged organelles and proteins within the cell [17]. Autophagy dysfunction is linked to neurodegenerative diseases where it causes an acceleration of the disease progression [20]. Studies have shown that autophagy inhibition also has a detrimental effect on the cardiovascular system. In addition, the evidence suggests that autophagy alternation is linked to several cardiovascular diseases such as atherosclerosis, cardiomyopathy, and myocardial infarctions [21]. This study provides evidence of the role of MnP, a redox-active compound and SOD mimetic, in mitigating the oxidative stress-induced disruption of autophagy in cardiomyocytes. Although various concentrations of rotenone treatment have previously been reported [22,23,24], we demonstrated the optimal dosage of rotenone to be 500 nM in inducing oxidative stress in cardiomyocytes. We also demonstrated that a rotenone treatment in the HL-1 cardiomyocyte led to increased ROS production, which caused the accumulation of 4-HNE-adducted proteins called aggresomes. Rotenone has been shown to have a pleiotropic effect in the cell. It causes the loss of mitohoncrial complex I activity and increases ROS generation, lipid peroxidation, and ER stress as well as suppressing autophagy and the depolymerization of microtubules [13,14,25,26,27]. We also showed that rotenone caused the suppression of complex I activity when treated with a 500 nM concentration for 24 h.

We demonstrated that this aggresome formation was due to the disruption of the autophagy process and that using an MnSOD mimetic could rescue the effect of rotenone. Furthermore, we showed that the specific part of the disruption of autophagy by ROS was caused by tubulin hyperacetylation upon rotenone treatment. The results of the study indicated that a rotenone treatment in HL-1 cardiomyocytes caused a dose-dependent rise in ROS production. These results were consistent with studies performed on other cell types such as HL-60 [12]. Similarly, ROS production increased in the ARPE-19 cell line following a rotenone treatment [11]. Our study revealed that increased ROS levels were rescued using the MnSOD mimetic MnTnBuOE-2-PyP^5+^ (MnP). ROS have a strong contribution to the pathogenesis of cardiovascular disease; antioxidant use has been shown to have a therapeutic potential in treating cardiovascular diseases. Specifically, several mitochondrial-based antioxidants have been popular in treating heart disease because the mitochondria are the major site of ROS production [5]. Different types of metal complexes containing Mn with MnSOD-like activity have been developed over the years. Among them, MnTnBuOE-2-PyP^5+^ (MnP) shows promise as a potential pre-treatment for ROS-induced damage due to its lipophilicity and less toxic profile when compared with other manganese (Mn)-based mimetics [28]. MnP is currently undergoing early phase clinical trials to reduce oxidative damage caused by radiation and chemotherapy. Furthermore, MnP has been tested and shown to mitigate oxidative damage in several cell types [29,30]. MnP reduces ROS levels either directly or indirectly through the inhibition of NF-kB or the activation of the Nrf2 signaling pathways [15,30].

Lipid peroxidation occurs whenever ROS react with polyunsaturated fatty acids, leading to a reactive aldehyde known as 4-hydroxynonenal (4-HNE). 4-HNE forms a covalent adduct that leads to the modification of nucleophilic functional groups such as DNA, proteins, and lipids responsible for carrying different signals inside the cell [31]. 4-HNE is a double-edged sword; it acts as a signaling molecule for cell survival at lower concentrations and causes cell death when present in higher concentrations. 4-HNE mainly reacts with the amino acids cysteine, histidine, and lysine and is involved in the pathogenesis of various pathologies, including neurodegenerative disease, chronic obstructive pulmonary disease, cardiovascular disease, cancer, cataracts, alcoholic liver disease, and inflammatory diseases [32]. Rotenone is a lipophilic molecule that inhibits complex I of the electron transport chain, causing an increase in mitochondrial ROS [33]. We showed that a treatment with rotenone increased the mitochondrial ROS in HL-1 cells. ROS lead to the formation of 4-HNE aggresomes, which we demonstrated were rescued by using the MnSOD mimetic, MnTnBuOE-2-PyP^5+^. This impact of MnP-driven rescue supports the data that rotenone causes an increase in mitochondrial ROS [34,35]. In our study, 4-HNE protein aggresomes co-localized with ubiquitin, HDAC6, and γ-tubulin. This revealed that 4-HNE aggresomes collected the misfolded proteins that participate in the autophagy process.

Cardiac injury and heart failure have been linked to the disruption of the autophagy process, sparking interest in targeting autophagy to treat heart disease. In our study, a central protein involved in autophagosome expansion, LC3 [36], was seen within the 4-HNE aggresomes. This suggested a clear link between the accumulation of 4-HNE within the cells and the autophagy process. We wondered whether a 4-HNE accumulation upon rotenone treatment was due to the disruption of the autophagy process or the failure of the autophagy stimulation. To study this, we overexpressed cells with Tandem fluorescent-tagged GFP-RFP LC3, which resulted in more significant numbers of autophagosomes than autolysosomes following a rotenone treatment. Once we knew that the oxidative stress disrupted the autophagy process, we hypothesized that using a mitochondrial-based SOD mimetic would effectively rescue autophagy flux in the cells challenged with rotenone. However, when pre-treated with MnP, the number of autolysosomes was equal to that of the autophagosomes. We further verified that rotenone disrupted autophagy flux by using bafilomycin, a known inhibitor of lysosomes. We observed no further increase in LC3-II expression by using bafilomycin in the rotenone-treated group. However, in the control and MnP-treated cells, the expression of LC3-II was remarkably increased following the bafilomycin treatment. This indicated that rotenone disrupted the autophagy process at the final step, which was prevented by MnP. We also discovered that the dysfunctional autophagy process was not due to a problem with the lysosomal function because there was no change in the lysosomal pH between the treatment groups.

Once we verified the effect of rotenone on ROS production within the cell, we wanted to discover the mechanism by which rotenone disrupts the autophagy process. The final step of the autophagy process is a fusion of the autophagosome with a lysosome, resulting in the degradation of unwanted material by the action of the hydrolases present in the lysosome. This fusion is thought to be achieved by microtubules, dynamic structures within the cell that constantly undergo polymerization and depolymerization to achieve stability within the cell [37]. Several studies have suggested that microtubules participate in the different stages of the autophagy process [38,39]. In addition, microtubules undergo various post-translational modifications such as acetylation on lysine residues [40]. Hyperacetylation has been implicated in the disruption of intracellular trafficking [8]. In our body of work, we demonstrated that rotenone-induced ROS lead to an increase in tubulin acetylation and the subsequent disruption of autophagy flux. This result suggested that the disruption of autophagy could be due to tubulin hyperacetylation, which prevents the fusion of the autophagosome with the lysosome, causing the incomplete clearance of aggresomes from the cells. Therefore, we hypothesized that the cytotoxic effect of rotenone on the cell was due to the failure of the autophagy process. We used rapamycin and 3-MA in cell cultures challenged with rotenone to support this hypothesis. Rapamycin activated autophagy by inhibiting mTOR and 3-MA, an autophagy inhibitor [41].

**Figure 8:**
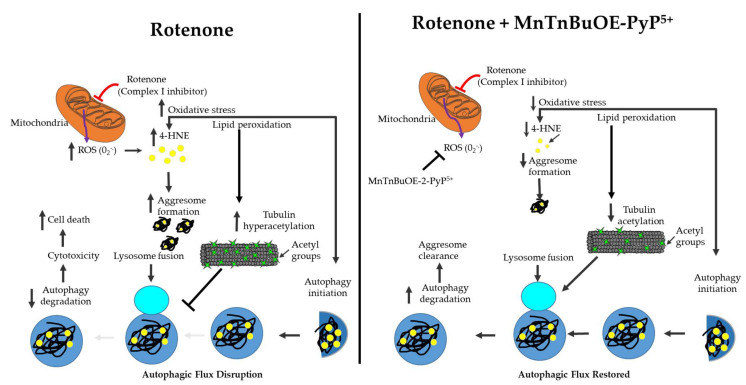
Graphical summary of the present study. Rotenone, a complex I inhibitor, induces oxidative stress in HL-1 cells by generating superoxide radicals. This, in turn, leads to the accumulation of 4-HNE aggresomes by the disruption of autophagy flux. This is partly due to an impairment in autophagosome–autolysosome fusion caused by tubulin hyperacetylation. Treatment with SOD mimetic stimulated autophagy flux, leading to decreased accumulation of 4-HNE aggresomes.

The addition of rapamycin was shown to aggravate cell death challenged by rotenone whereas 3-MA decreased cell death. These results suggested that the autophagosome accumulation was enough to cause cell toxicity. This was supported by several studies showing cardiomyocyte protection by the stimulation of autophagy in several heart pathologies [42,43]. The graphical representations summarize the pathological mechanism by which rotenone causes cytotoxicity in HL-1 cells and demonstrate how MnSOD mimetic MnTnBuOE-2-PyP5+ (MnP) can rescue its effects (Figure 8).

## 4. Materials and Methods

### 4.1. Cell Culture

HL-1 cells were purchased from Millipore Sigma and cultured on 100 mm plates. The cell culture plates were coated with gelatin/fibronectin and grown in Claycomb media (Millipore Sigma, St. Louis, MO, USA) supplemented with the following: 10% fetal bovine serum, 1% norepinephrine, 1% penicillin/streptomycin, and 1% L-glutamine. The cells were cultured in a humified incubator at 37 °C and 5% CO2. We used 8- to 10-week-old male C57BL/6 mice (Jackson Laboratory, Bar Harbor, ME, USA) for the isolation of adult cardiac myocytes in the current study, as recently shown by our group [44]. To identify the effect of rotenone in HL-1 cells, rotenone (Millipore Sigma) dissolved in dimethyl sulfoxide (DMSO) and diluted in starvation media to produce a working concentration was used to treat the cells for 24 h. The cells were pre-treated with MnP at a concentration of 20 µM for 24 h before the rotenone treatment.

### 4.2. Determination of Intracellular Superoxide

The cells were grown in six-well plates until they reached a confluence. The cells were stimulated with rotenone (500 nM, 24 h) or MnTnBuOE-2-PyP^5+^ (20 µM, 24 h) followed by rotenone (500 nM, 24 h). The cells were incubated in 10 µM of dihydroethidium (DHE) during the final 30 min of treatments. After washing twice with ice-cold PBS, the cells were centrifuged in 1 mL DPBS. The pellets obtained after centrifugation were lysed in 0.1% Triton X 100 and centrifuged at 10,000 *g* for 5 min at 4 °C. The supernatant was transferred into a tube containing an equal amount of 0.2 M HClO_4_ in MeOH. After centrifugation, the supernatant was transferred into a tube containing an equal amount of 1 M phosphate buffer. The supernatant obtained after centrifugation for 5 min × 12,000 *g* at 4 °C was subjected to an HPLC reading.

### 4.3. Complex I Activity Assay

The complex I activity was spectrophotometrically measured, as described previously [45]. The cells were grown on 100 mm discs until they reached a confluence. Following the treatment with rotenone (500 nM, 24 h), a cell pellet was obtained and flash-frozen in liquid nitrogen. A total of 100 µg protein was mixed in a reaction mixture containing a potassium phosphate buffer (0.5 M, pH 7.5), fatty acid-free BSA (50 mg/mL), KCN (10 mM), NADH (10 mM), and ubiquinone 1 (10 mM). A spectrophotometer (DeNovix, Wilmington, DE, USA) was used to measure NADH oxidation before and after the addition of rotenone (10 μmol/L).

### 4.4. Determination of Mitochondrial ROS

Mitochondrial ROS were determined with MitoSOX (Thermo Fisher Scientific, Waltham, MA, USA). A total of 5 µM MitoSOX was prepared in complete media and incubated for 20 min at 37 °C in a 5% CO_2_ incubator. The cells were washed twice with PBS. A fluorescence microscope determined the MitoSOX fluorescence intensity.

### 4.5. Immunofluorescence

The HL-1 cells were fixed in 4% formaldehyde for 20 min and were washed with PBS three times. The cells were permeabilized with 0.2% Triton for 10 min. The cells were blocked with 10% goat serum for 1 h following permeabilization. The following primary antibodies were added and incubated overnight: acetylated tubulin (Millipore Sigma); HDAC6 (Cell Signaling, Danvers, MA, USA); 4-Hydroxynonenal (Abcam, Cambridge, MA, USA); LC3 (Cell Signaling); ϒ-tubulin (Abcam); and ubiquitin (Abcam). AlexaFluor 488 goat anti-mouse (ThermoFisher), AlexaFluor 488 goat anti-rabbit (ThermoFisher), or AlexaFluor 594 goat anti-rabbit (ThermoFisher) secondary antibodies were added for 1 h at room temperature. The cell nuclei were stained with DAPI in a VECTASHIELD Mounting Medium (H-1800, Vector Laboratories) and imaged using fluorescence microscopy.

### 4.6. Western Blot

The HL-1 cells were washed thrice with ice-cold PBS and then collected after cell scraping in a RIPA lysis buffer supplemented with a proteinase cocktail and PhosStop (a phosphatase inhibitor). The cell suspension was centrifuged at 14,000 rpm for 15 min at 4°C. Equal amounts of protein were loaded into 10% and 15% SDS-PAGE gels. The blots were transferred onto an Immobilon transfer membrane followed by 2 h of blocking with 0.1% Casein (ThermoFisher Scientific) in PBS. The immunoblots were incubated overnight with the following antibodies: LC3 (Cell Signaling Technology) (1:1000); acetylated tubulin (Millipore Sigma) (1:5000); Beclin-1 (Abcam) (1:1000); β-Actin (Cell Signaling Technology) (1:1000); and GAPDH (Cell Signaling Technology) (1:1000) as a loading control. A Li-COR secondary antibody was used. The bands were detected and quantified using a Li-COR Odyssey system.

### 4.7. Real-Time Polymerase Chain Reaction (RT-PCR)

Total RNA was isolated from the cell culture dishes using a Purelink RNA mini kit (Invitrogen, San Diego, CA, USA). The cDNA was synthesized using TaqMan Reverse Transcription Reagents (Applied Biosystems, ThermoFisher Scientific, Waltham, MA, USA) in a thermal cycler. Primers for ATG-5 and PIK3C3 (Applied Biosystems) were used. The relative expression of the target gene was measured using the 2^−ΔΔct^ method.

### 4.8. Transfections

The cells were plated on an SPL 8-well cell culture chamber 24 h prior to transfection. The cells were transfected with the desired adenovirus GFP-LC3. The cells were incubated for 2 h before the transfection mixture was removed and replaced with a completely fresh medium. The cells were incubated for 24 h to allow for the protein expression. Following the protein expression, the cells were either treated with rotenone alone or pre-treated with MnP prior to the treatment with rotenone. Dr. Md Shenuarin Bhuiyan kindly provided GFP-LC3 (Louisiana State University Health Sciences Center, Shreveport).

### 4.9. Autophagosome Maturation

The HL-1 cells were transfected with tandem fluorescent-tagged LC3 and incubated for 2 h to detect autophagy flux. The transfection mixture was removed and replaced with fresh media. The cells were incubated for 24 h to allow for the expression of proteins. Dr. Md Shenuarin Bhuiyan kindly provided LC3 (Louisiana State University Health Sciences Center, Shreveport).

### 4.10. Intralysosomal pH

For the lysosomal pH, LysoTracker^®^ Deep Red (ThermoFisher Scientific) was diluted in warm media to a final concentration of 60 nM and incubated at 37 °C for 1 h. After 1 h, the cells were washed twice with warm PBS and imaged with a fluorescent microscope.

### 4.11. Cell Viability Assay

Following a treatment with rotenone (500 nM), 3-MA (autophagy inhibitor 5 mM), rapamycin (autophagy inducer 20 nM), and the control cells were trypsinized and centrifuged at 1000 rpm for 6 min to obtain a cell pellet. The cell pellets were re-suspended in complete media for cell counting. One part of the cell suspension was mixed with 0.4% trypan blue and collected to visualize the cell under a bright-field microscope. The cell viability was measured by the trypan blue exclusion method.

### 4.12. Statistical Analysis

The data were analyzed as the mean + standard error of the mean (SEM) using GraphPad Prism software (Version 7, GraphPad, San Diego, CA, USA). To compare the results between two groups, the Student’s *t*-test was used. The data were tested for multiple comparisons with a one-way ANOVA followed by a Tukey test or a two-way ANOVA. In addition, the Bonferroni test was performed for the normally distributed data. The results were considered to be statistically significant when *p* < 0.05.

## 5. Conclusions

Our study demonstrated that rotenone-induced ROS led to the hyperacetylation of microtubules and subsequent disruption of the autophagy process. The disruption was followed by an accumulation of autophagosomes within the cell, causing deleterious effects and subsequent cytotoxicity. Through our study, we found that MnP could rescue the effect of rotenone by suppressing the cellular oxidative stress (either directly via scavenging the reactive species or indirectly via oxidatively modifying the signaling proteins) and thus prevent tubulin hyperacetylation and the subsequent accumulation of aggresomes. This study provides an insight into disease pathways that involve increased mitochondrial ROS and the disruption of the autophagy process.

## Data Availability

Not applicable.
